# Supplementary material for: Antagonistic Activity of Streptomyces alfalfae 11F against Fusarium Wilt of Watermelon and Transcriptome Analysis Provides Insights into the Synthesis of Phenazine-1-Carboxamide
Source: Plants (Basel). 2023 Nov 8;12(22):3796. doi: 10.3390/plants12223796 (PMC10675820; doi:10.3390/plants12223796)
Supplement: Supplementary file 1 [file plants-12-03796-s001.zip › plants-2619998-supplementary.pdf]

Supplementary material

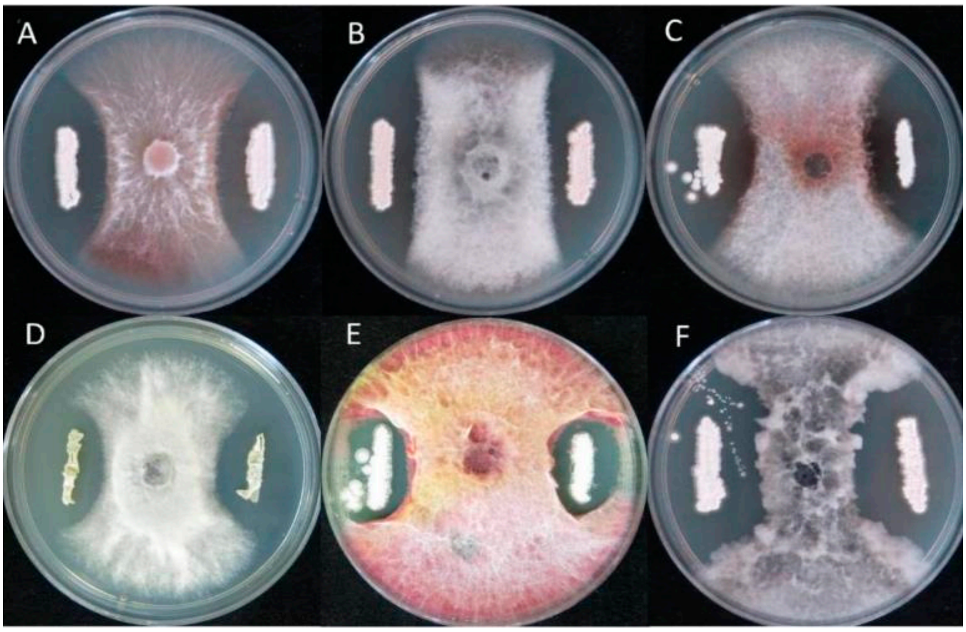

Figure S1. Antifungal activity of strain 11F against six pathogens. (**A**) *F. oxysporum* f. sp. *niveum*. (**B**) *S. turcica*. (**C**) *F. solani*. (**D**) *B. cinerea*. (**E**) *F. graminearum*. (**F**) *B. dothidea*.

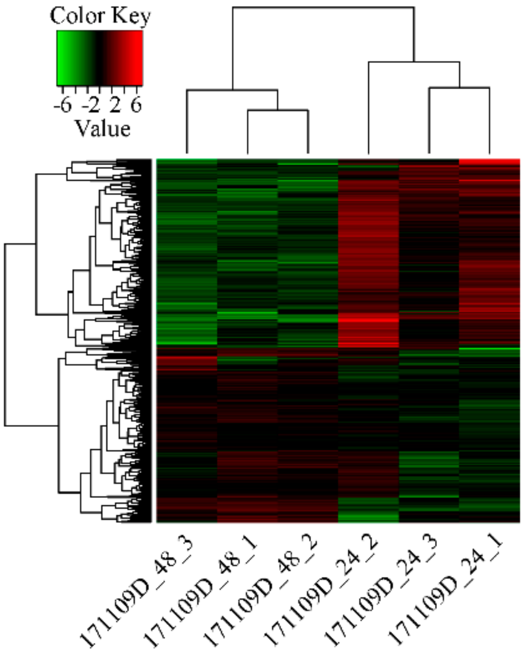

Figure S2. Expression patterns of the DEGs between Case 1 and Case 2.

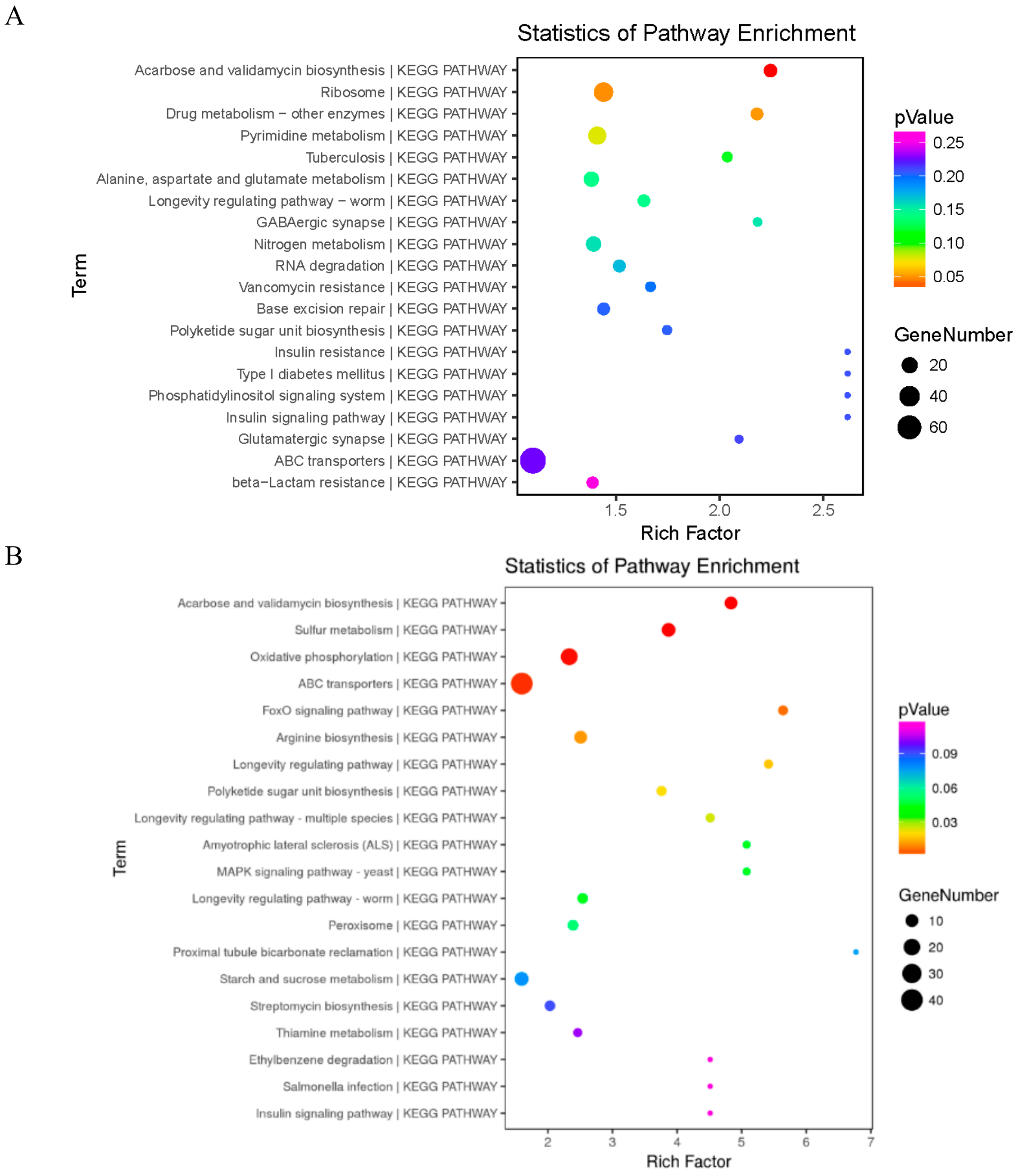

Figure S3. Top 20 enriched KEGG pathways. (**A**) Control *vs* Case 2 comparison. (**B**) Case 1 *vs* Case 2 comparison. The Rich Factor refers to the ratio of the number of differentially expressed transcripts in the pathway entry to the total number of transcripts in the pathway entry. Increases in the Rich Factor reflect increases in the degree of enrichment. The dot size indicates the number of DEGs assigned that particular term, whereas the dot color indicates the *p*-value.

A

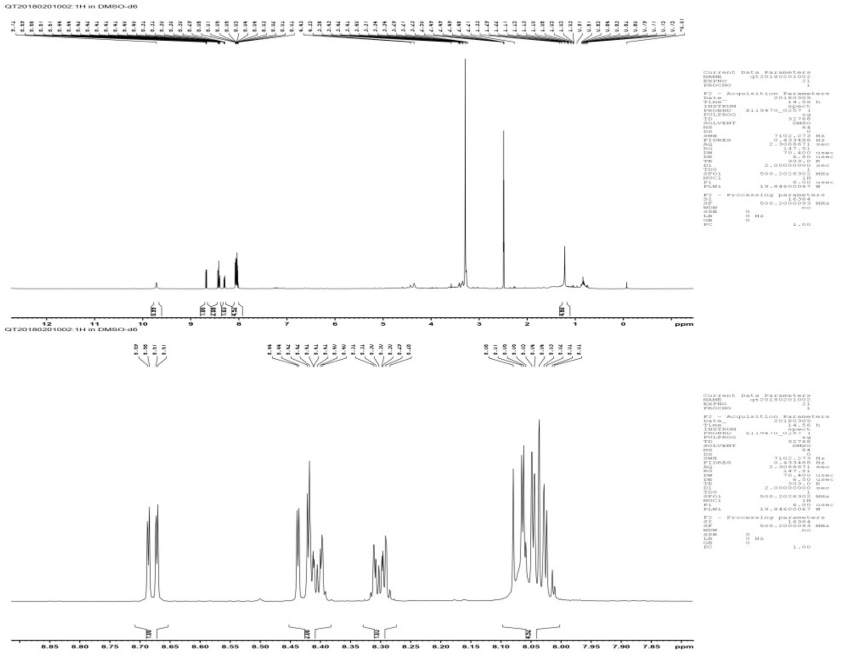

B

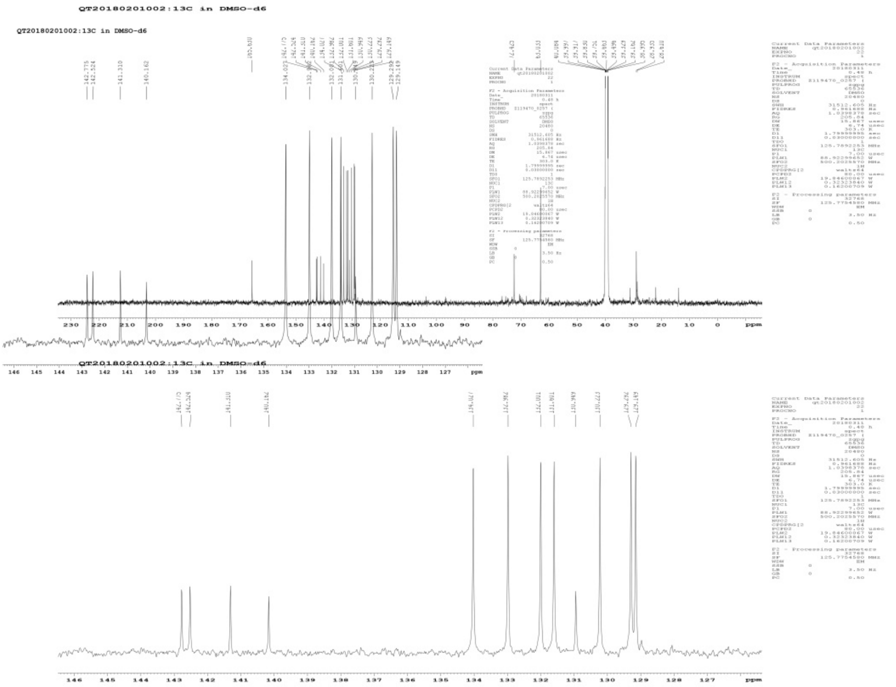

Figure S4. ^{1}H NMR (**A**) and ^{13}C NMR (**B**) of the phenazine compound (in DMSO-d6)

Table S1. Strain 11F inhibitory effects on various fungi

Pathogen	Inhibition diameter	Inhibition rate（%）
F. oxysporum f. sp. *niveum*	30 ± 2.31	64.70
S. turcica	39 ± 2.00	54.17
F. solani	48 ± 3.61	43.52
B. cinerea	50 ± 3.05	41.17
F. graminearum	40 ± 2.08	52.94
B. dothidea	31 ± 2.00	63.52

Table S2. Details regarding the RNA-seq data

Sample	Raw reads number	Raw bases	Clean reads number	Clean rate(%)	GC(%)	Q20(%)	Q30(%)
171109D_24_1	15884350	2356344297	15337414	96.56	58	98.5	95.4
171109D_24_2	14898594	2222789172	14205810	95.35	68	98.05	94.17
171109D_24_3	15369888	2284379201	14851614	96.63	61	98.46	95.25
171109D_48_1	15651094	2313738464	15045242	96.13	58	98.47	95.35
171109D_48_2	13565412	2004702463	13069544	96.34	59	98.46	95.28
171109D_48_3	16731524	2480653375	16154234	96.55	61	98.44	95.2
171109D_6_1	16491404	2458547908	15834006	96.01	64	98.21	94.57
171109H_6_2	13839518	2066130098	13164796	95.12	64	98.09	94.29
171109H_6_3	16502726	2453384678	15838784	95.98	62	98.28	94.78

Raw read number: the number of raw data sequences (reads); Raw bases: the total number of bases in the raw data; Clean read number: the number of filtered data sequences (reads); Clean bases: the total number of bases after filtering; Clean rate (%): the total number of reads after filtering as a percentage of the total number of reads in the original data; Q20 (%): the number of bases with a quality value greater than 20 as a percentage of the total number of clean bases; Q30 (%): the number of bases with a quality value greater than 30 as a percentage of the total number of clean bases.

Table S3. Mapping of the clean reads to the reference genome

Samples	Total reads	Mapped reads	Uniquely mapped reads	Multiple mapped reads
171109D_24_1	15337414(100.00%)	13636312(88.91%)	12495511(91.63%)	1140801(8.37%)
171109D_24_2	14205810(100.00%)	13938198(98.12%)	13589269(97.50%)	348929(2.50%)
171109D_24_3	14851614(100.00%)	14245527(95.92%)	13947432(97.91%)	298095(2.09%)
171109D_48_1	15045242(100.00%)	12077527(80.27%)	9572866(79.26%)	2504661(20.74%)
171109D_48_2	13069544(100.00%)	12589489(96.33%)	11980742(95.16%)	608747(4.84%)
171109D_48_3	16154234(100.00%)	15533734(96.16%)	14941632(96.19%)	592102(3.81%)
171109D_6_1	15834006(100.00%)	15346023(96.92%)	15052779(98.09%)	293244(1.91%)
171109H_6_2	13164796(100.00%)	12897613(97.97%)	10922347(84.69%)	1975266(15.31%)
171109H_6_3	15838784(100.00%)	15369356(97.04%)	12031528(78.28%)	3337828(21.72%)

Total reads: the number of filtered data sequences; Mapped reads: the number of reads mapped to the reference genome sequence (the proportion of such reads as a percentage of the total number of reads); Uniquely mapped reads: the number of reads mapped to a unique location of the reference genome sequence (the proportion of such reads as a percentage of the number of mapped reads); Multiple mapped reads: the number of reads mapped to multiple locations in the reference genome sequence (the proportion of such reads as a percentage of the number of mapped reads).

Table S4. Detailed information for the selected genes for qPCR

ID	Description
A7J05_RS03465	XRE family transcriptional regulator
A7J05_19090	phosphate ABC transporter substrate-binding protein PstS
A7J05_03770	phosphate ABC transporter substrate-binding protein PstS
A7J05_10310	DegT/DnrJ/EryC1/StrS aminotransferase
A7J05_31935	GNAT family N-acetyltransferase
A7J05_19075	phosphate ABC transporter ATP-binding protein
A7J05_03610	N-acetylmuramoyl-L-alanine amidase
A7J05_03600	aminoglycoside phosphotransferase family protein
A7J05_19080	phosphate ABC transporter permease PtsA
A7J05_08410	AraC family transcriptional regulator
A7J05_03975	ergothioneine biosynthesis protein EgtC
A7J05_09920	amino acid acetyltransferase
A7J05_11495	ABC transporter ATP-binding protein
A7J05_29000	FadR family transcriptional regulator
A7J05_09325	polysaccharide biosynthesis protein
A7J05_31855	TerD family protein
A7J05_11670	MFS transporter
A7J05_15660	ABC transporter substrate-binding protein
A7J05_35185	ABC transporter permease
A7J05_11930	ABC transporter ATP-binding protein

Table S5. Prediction of secondary metabolites in strain 11F using antiSMASH database

Cluster type	Start gene	End gene	Most similar known cluster	（%） Similarity
butyrolactone-like-pks-nrps	51512	33151389	Microtermolide A	46
terpene	177378	199295	Lavendiol	6
t1pks-nucleoside-nrps	316214	485864	Nystatin	36
transatpks-nrps	597379	695568	Oxazolomycin	84
nrps-like	759075	798968	Hygromycin B	12
t3pks-nrps-betalactone	820218	888280	Lomaiviticin	6
indole-transatpks-nrps	940834	1004891	Cinnabaramide	18
t3pks-nrps	1056258	1144347	Balhimycin	43
thiopeptide	1210965	1240305	Lavendiol	6
nrps	1305544	1348177	-	-
terpene	1508180	1533212	Hopene	84
nrps-like-hgIE-KS	1697106	1763315	-	-
t1pks	2100640	2145201	Calicheamicin	5
terpene	2269014	2289681	Geosmin	100
bacteriocin	2323434	23346271	-	-
terpene	2429713	2449228	Pentalenolactone	52
nrps	256766	2618409	Coelichelin	100
siderophore	2623445	2638428	Ficellomycin	3
nrps	3133645	3187074	Albaflavenone	36
terpene	3252815	3270943	Albaflavenone	100
t1pks-butyrolactone	3421226	3464976	Tetronasin	9
nrps-ladderane	4115633	4182686	Ishigamide	100
melanin	4275008	4285445	Istamycin	5
t1pks-nrps	6098225	6147146	GE81112	7
ectoine	6585365	6595769	Ectoine	100
nrps	7420986	7469702	Mirubactin	50
t3pks	7597881	7636578	BE-14106	14
terpene	7702912	7727000	Isorenieratene	100
t1pks-nrps	7800651	7858021	GE81112	10
bacteriocin-indole-t1pks-terpene-nrps	7861674	7974735	A-503083	7
t1pks-nrps-like	8020578	8065515	Herboxidiene	5
other-CDPS	8134917	8175747	Lavendiol	6
t3pks	8221982	8263039	Alkylresorcinol	100
bacteriocin-nrps	8306025	8392219	Surugamide A / surugamide D	23
nrps	8467004	8525260	Vioprolide	16
butyrolactone	8560299	8571255	Griseoviridin / viridogrisein	5
